# Intravital Imaging of Adoptive T-Cell Morphology, Mobility and Trafficking Following Immune Checkpoint Inhibition in a Mouse Melanoma Model

**DOI:** 10.3389/fimmu.2020.01514

**Published:** 2020-07-22

**Authors:** Doreen Lau, Fabien Garçon, Anita Chandra, Laura M. Lechermann, Luigi Aloj, Edwin R. Chilvers, Pippa G. Corrie, Klaus Okkenhaug, Ferdia A. Gallagher

**Affiliations:** ^1^Cancer Research UK Cambridge Centre, Cambridge, United Kingdom; ^2^Department of Radiology, University of Cambridge, Cambridge, United Kingdom; ^3^Laboratory of Lymphocyte Signaling and Development, The Babraham Institute, Cambridge, United Kingdom; ^4^Department of Medicine, University of Cambridge, Cambridge, United Kingdom; ^5^Department of Pathology, University of Cambridge, Cambridge, United Kingdom; ^6^Department of Nuclear Medicine, Addenbrooke's Hospital, Cambridge, United Kingdom; ^7^Department of Oncology, Addenbrooke's Hospital, Cambridge, United Kingdom

**Keywords:** intravital imaging, adoptive T-cell therapy, immune checkpoint inhibitors, immunocompetent, melanoma

## Abstract

Efficient T-cell targeting, infiltration and activation within tumors is crucial for successful adoptive T-cell therapy. Intravital microscopy is a powerful tool for the visualization of T-cell behavior within tumors, as well as spatial and temporal heterogeneity in response to immunotherapy. Here we describe an experimental approach for intravital imaging of adoptive T-cell morphology, mobility and trafficking in a skin-flap tumor model, following immune modulation with immune checkpoint inhibitors (ICIs) targeting PD-L1 and CTLA-4. A syngeneic model of ovalbumin and mCherry-expressing amelanotic mouse melanoma was used in conjunction with adoptively transferred OT-1^+^ cytotoxic T-cells expressing GFP to image antigen-specific live T-cell behavior within the tumor microenvironment. Dynamic image analysis of T-cell motility showed distinct CD8^+^ T-cell migration patterns and morpho-dynamics within different tumor compartments in response to ICIs: this approach was used to cluster T-cell behavior into four groups based on velocity and meandering index. The results showed that most T-cells within the tumor periphery demonstrated Lévy-like trajectories, consistent with tumor cell searching strategies. T-cells adjacent to tumor cells had reduced velocity and appeared to probe the local environment, consistent with cell-cell interactions. An increased number of T-cells were detected following treatment, traveling at lower mean velocities than controls, and demonstrating reduced displacement consistent with target engagement. Histogram-based analysis of immunofluorescent images from harvested tumors showed that in the ICI-treated mice there was a higher density of CD31^+^ vessels compared to untreated controls and a greater infiltration of T-cells towards the tumor core, consistent with increased cellular trafficking post-treatment.

## Introduction

Adoptive T-cell therapy (ATC) is emerging as a promising approach for the treatment of solid cancers (1, 2). Tumor-infiltrating lymphocytes (TIL) and chimeric antigen receptor (CAR) T-cells are currently undergoing Phase I/II clinical trials for treatment of solid malignancies (2, 3). However, ATC presents significant challenges for routine clinical use due to variable patient response to therapy and the requirement for a high specificity to tumor-associated antigens (4, 5). T-cell infiltration into normal tissues after systemic administration raises the possibility for off-target and dose-limiting toxicities (4–6). Furthermore, T-cell trafficking and infiltration into solid tumors is limited by physical barriers such as the stromal network and extracellular matrix surrounding the tumor cells. Regional delivery of T-cells via intratumoral injection has been proposed as a more targeted method to overcome these physical barriers, but remains technically challenging as not all tumors are easily accessible and it is difficult to reproducibly distribute the injected cells within the tumor (7, 8).

In addition, the efficacy of ATC is limited by hostile microenvironmental factors such as hypoxia and metabolite depletion caused by rapidly growing tumors, the presence of immunosuppressive cell types such as cancer-associated fibroblasts, regulatory T-cells, and myeloid-derived suppressor cells, many of which express ligands for immune checkpoint receptors. Synergistic combination of ATC with other targeted therapies to alter the tumor microenvironment may overcome these limitations (9–11).

Monoclonal antibodies targeting the cytotoxic T-lymphocyte antigen-4 (CTLA-4) and programmed cell death 1 (PD-1/PD-L1) checkpoint pathways are used in the clinic to treat several solid tumors such as metastatic melanoma and non-small cell lung carcinoma (12–16). Immune checkpoint inhibitors may improve the efficacy of ATC by releasing constitutive inhibition on T-cell function through depletion of subpopulations of immunosuppressive cells within the tumor microenvironment via F_c_-mediated antibody-dependent cellular cytotoxicity (ADCC) mechanisms (17). Several recent clinical trials have demonstrated the safety and efficacy of combining ATC with CTLA-4 and PD-1/PDL1 targeting strategies: for example, using anti-PD-1 as an immune booster after intrapleural CAR T-cell administration in patients with mesothelin-positive malignant pleural disease (18), novel third-generation CAR T-cells with the capability for expressing and secreting CTLA-4 and PD-1 antibodies into the tumor microenvironment (19), and CRISPR/Cas9 PD-1 knockout CAR T-cells (20). Many of these ATC methods utilize prior lymphodepletion to reset the regulatory components of the immune system and promote the engraftment and long-term persistence of transferred T-cells. Whilst this enhances the anti-tumor efficacy of transferred T-cells, it is potentially toxic for patients (21, 22). *Ex vivo* T-cell stimulation and expansion before autologous administration has also been reported to cause massive cytokine release, which necessitates intensive monitoring of patients (23). Little is known about how combined treatment with immune checkpoint inhibitors affects immunosuppression within the solid tumor microenvironment or whether it modulates adoptive T-cell function and behavior *in vivo*. Flow cytometry and other *in vitro* assays are limited, as they do not provide information on the spatial and temporal heterogeneity of T-cell response within living organisms, a hallmark of most tumors and a major driver of therapeutic failure. *In vivo* methods to dynamically study T-cell distribution, motility, and interaction with resident cellular subpopulations have the potential to reveal novel mechanisms of action as well as efficiently informing on the efficacy of treatments used in combination with these cell therapies. In particular, *in vivo* imaging can reveal spatial and temporal heterogeneity at high resolution which is difficult with other approaches. There is currently an unmet need for novel imaging approaches to study adoptive T-cell motility within the solid tumor microenvironment, as well as how immune modulation with checkpoint inhibitors can affect T-cell infiltration and migration patterns.

Intravital imaging using multiphoton microscopy is an example of an imaging tool that can be used for the direct visualization and characterization of cell behavior and spatiotemporal dynamics of physiological processes within living organisms. The technique has been used for studying various aspects of innate and adaptive immune responses to cancer, infection and inflammatory disorders at single-cell resolution (24, 25), and is complementary to macroscopic imaging techniques for *in vivo* cell tracking, such as positron emission tomography (PET), single-photon emission computed tomography (SPECT) and magnetic resonance imaging (MRI) (26–28).

In this study, intravital imaging was used to evaluate adoptive T-cell morphology, mobility and trafficking in the solid tumor microenvironment of immunocompetent mice. A syngeneic model of ovalbumin-expressing mouse melanoma with adoptively transferred green-fluorescent OT-1^+^ cytotoxic T-cells was used to study antigen-specific T-cell behavior in skin-flap tumors. This experimental setup used a widely available transgenic model to demonstrate the feasibility of studying T-cell mobility and behavior *in situ*. Treatment with immune checkpoint inhibitors was used as a model system to demonstrate the potential applications of this methodology and the work has revealed novel morpho-dynamic characteristics of ATC behavior following therapy with ICIs. This approach could be used to investigate and screen novel combinational ATC-drug regimens in the future, which in turn could have important implications for designing clinical trials incorporating cell therapy and immune modulation.

## Materials and Methods

### Cell Culture

The mouse metastatic melanoma cell line B78ChOva-mCherry was obtained from Prof. Matthew F. Krummel (University of California, San Francisco). This transgenic line expresses the specific antigen chicken ovalbumin (OVA) peptide tagged to a mCherry reporter that can be recognized by antigen-specific OT-I^+^ T-cells (29). The line was generated via the transfection of B16 parental cells with a vector containing mCherry and chicken ovalbumin genes intercalated with two self-cleavable porcine teschovirus-1 2A (P2A) sequences. Cells tested negative for mycoplasma and rodent infectious agents and were cultured in RPMI-1640 media containing *L*-glutamine (Gibco®, Thermo Fisher Scientific, UK) with 10% heat-inactivated fetal bovine serum (LabTech, UK) and 100 U/mL penicillin-streptomycin (Invitrogen, USA) in a humidified atmosphere of 5% CO_2_ at 37°C.

### Mouse Models

All animal procedures were approved by the Animal Welfare and Ethics Review Committees of the University of Cambridge and the Babraham Institute in Cambridge and were carried out in accordance with the United Kingdom Home Office's Guidance on the Operation of Animals (Scientific Procedures) Act 1986.

OT-I x GFP mice were generated by crossing OT-1 mice (30) with UBI-GFP mice (31) in the C57BL/6 background and the offspring were genotyped using the following PCR primers:

OT-I T-cell Receptor (TCR) Vα2 chain

Forward 5′-CAGCAGCAGGTGAGACAAAGT-3′ and

Reverse 5′-GGCTTTATAATTAGCTTGGTCC-3′

GFP

Forward 5′-AAGTTCATCTGCACCACCG-3′ and

Reverse 5′-TCCTTGAAGAAGATGGTGCG-3′.

Female mice (12–14 weeks old) that were positive for both OT-I and GFP were used as T-cell donors into recipient female C57BL/6 mice (8–10 weeks old).

### Generation of a Syngeneic Mouse Melanoma Model for Immunotherapy and T-Cell Intravital Imaging

Syngeneic mouse melanoma models for immunotherapy were established by subcutaneous injection of B78ChOva-mCherry cells (1.5 × 10^5^ cells in 100 μl PBS) in the left flank of C57BL/6 mice on Day 0. Tumors were palpable by Day 10.

Naïve OT-I^+^ CD8^+^ GFP^+^ T-cells were isolated and purified from donor OT-I x GFP transgenic mice (non-tumor bearing females) for adoptive transfer into recipient female tumor-bearing mice with 10-day old tumors. This was conducted based on the following procedures:

The peripheral and mesenteric lymph nodes were first harvested from donor OT-I x GFP mice into MACS buffer (PBS containing 0.5% bovine serum albumin and 2 mM EDTA). Lymph nodes pooled from a single donor OT-I x GFP mouse produced sufficient T-cells for adoptive transfer into 10 mice. In each trial, we pooled CD8^+^ T-cells from the lymph nodes of 2 donor mice for injection into 18 recipient tumor-bearing mice, which were then randomly assigned into different treatment cohorts.

1. The lymph nodes were extruded through a 40 μm cell strainer (Falcon^TM^, Thermo Fisher Scientific, UK) into a petri dish, rinsed with MACS buffer and centrifuged at 1,500 rpm for 5 min. The cells were then resuspended at 10^8^ cells/ml in MACS buffer.
2. CD8^+^ T-cells were enriched by the negative selection magnetic sorting method with fluorescein isothiocyanate (FITC)-conjugated antibodies (Biolegend, USA) at 2.5 μg/ml as shown in Table 1.
3. The cell suspension was incubated with FITC-conjugated antibodies in 1 ml MACS buffer at 4°C for 30 min.
4. Incubation of the cell suspension was terminated by adding 10 ml MACS buffer to the antibody labeled cells. The cells were then pelleted by centrifugation at 1,200 rpm for 7 min.
5. 10^7^ cells labeled with antibodies were incubated with anti-FITC microbeads (Miltenyi Biotec, Germany) at 1:10 dilution on a rotator at 4°C for 20 min.
6. Incubation of cells was terminated by adding 10 ml MACS buffer to the antibody labeled cells. The cells were then pelleted by centrifugation at 1,500 rpm for 5 min and resuspended in 1 ml MACS buffer.
7. Elution of the cell suspension was carried out through a LS column set up on a MidiMACS^TM^ Separator (Miltenyi Biotec, Germany). The eluent (isolated CD8^+^ T-cells) were collected and the number of cells were counted.
8. Purity of the OT-I^+^ CD8^+^ GFP^+^ T-cells were verified by flow cytometry (BD LSRFortessa^TM^ analyzer, BD Biosciences, USA) with CD4-PE (clone GK1.5), CD8a-APC (clone 53–6.7) and TCRVα2-PerCPCy5.5 (OT-I; clone B20.1) antibodies (Biolegend, USA), as well as the expression of GFP.
9. 10^5^ T-cells were adoptively transferred into each syngeneic tumor mouse by intravenous injection.

**Table 1:** List of FITC-conjugated antibodies for isolation and purification of CD8^+^ T-cells from lymph nodes of donor OT-I x GFP mice using the negative selection magnetic sorting method.

**Antibodies**	**Clone**	**Removal of non-target cells**
CD4	H129.19	CD4 T-cells
CD19	MB19-1	B-cells
CD49b	DX5	NK cells
MHCII i[Ab]	AF6-120.1	Antigen-presenting cells
CD25	PC61	Activated T-cells
CD69	H1.2F3	Activated T-cells

### Treatment With Immune Checkpoint Inhibitors

To evaluate the experimental approach for intravital imaging of adoptive T-cells in the context of cancer immunotherapy using immune checkpoint inhibitors, treatment was administered intravenously into mice with established tumors from Day 13 after the adoptive T-cell transfer experiment every 2–3 days over the course of 1 week into three treatment cohorts with *n* = 18 mice per group from three independent experiments: (1) vehicle control; (2) anti-PD-L1 monotherapy; and (3) anti-CTLA-4 + anti-PD-L1 (10 mg/mL each; anti-CTLA-4: clone 9H10; anti-PD-L1: clone 10F.9G2; InVivoMAb^TM^, Bio X Cell, USA).

Tumor growth was monitored from Day 11 onwards by caliper measurements every 2–3 days, and volumes were calculated using an ellipsoid formula for best estimation of the tumor mass: *volume (mm*^3^*)* = *(*π*/6) x a x b x c*, where *a, b*, and *c* represent the three orthogonal axes of the tumor. Only mice with palpable tumors from Day 10 were included for adoptive transfer experiment, intravital imaging and subsequent analysis.

### Surgical Preparation of Skin-Flap Tumors for Intravital Imaging

Surgical preparation of the skin-flap tumors for intravital imaging was conducted based on the following procedures:

1. Anesthetization of the mouse was carried out in an induction chamber using 4% isoflurane with 21% oxygen and balance nitrogen as the carrier gas at a flow rate of 1.0 L/min. The time duration for anesthetization of the mouse was about 3–5 min.
2. Once the mouse was breathing deeply and slowly, it was transferred to a surgical stage equipped with a stereomicroscope. Surgical anesthesia was performed with a 1:1 mixture of Hypnorm (25 mg/kg) and Hypnovel (12.5 mg/kg) (VetaPharma Ltd, UK) delivered via intraperitoneal injection. To ensure that the mouse was properly anesthetized for the surgical procedure, a footpad pinch was performed to ensure no pedal withdrawal reflex.
3. The limbs of the mouse were then secured to the surgical platform using surgical tapes.
4. Hair was removed from the ventral surface around the imaging site using a shaver and the area was disinfected with 70% isopropanol wipes and betadine.
5. The surgical procedure began by first making a single ventral midline incision about 3 mm above the urethra to the xiphoid process using the first pair of sterile scissors and teethed forceps. Care was taken not to puncture or cut through the peritoneum.
6. Using a second pair of sterile scissors and serrated forceps, the skin was gently detached away from the peritoneal cavity, with the subcutaneous tumor and draining lymph node attached. Care was taken to ensure no damage to the major blood vessels to maintain blood perfusion.
7. The external surface of the skin-flap tumor was then secured to a deep-well heated perfusion chamber (Series 40 Perfusion Chamber, Warner Instruments, Harvard Bioscience Inc., USA) using veterinary-grade tissue adhesive glue (3M Vetbond, USA) such that the bulk of the tumor was on top and was seated flat once the mouse was placed on the microscope stage and was not hindered by the mouse body and mobility of its hind limbs. To minimize tissue dehydration, the exposed skin-flap tumor was submerged with surgical saline solution. The surrounding parts of the exposed tissue was covered with sterile gauze to protect against contamination.
8. Following surgery, the mouse was transferred immediately onto a portable microscope stage insert equipped with a heating pad (Harvard Apparatus, USA) to maintain its temperature at 37°C to prevent hypothermia. The mouse was gently secured to the stage insert using surgical tapes.
9. Both the mouse on the stage insert and the perfusion chamber were transferred onto a microscope stage covered with sterile gauze. Care was taken to ensure that the tumor was correctly positioned on top and in the center of the imaging port of the microscope stage. The perfusion chamber was secured to the microscope stage with lab tapes to minimize motion artifacts during imaging. This was followed by the attachment of the inlet and outlet ports of the perfusion chamber to a vacuum pump system (VACUSAFE, Integra Biosciences, UK) for constant bathing of the exposed skin-flap tumor with warm oxygenated saline during imaging.
10. For continuous anesthesia and rehydration of the mouse during imaging, an intraperitoneal line was inserted with a winged infusion set attached to a 1 ml syringe containing 100 μl saline. The animal was injected with 50 μl of saline and 1:1 Hypnorm/Hypnovel mix at 1 h intervals until the end of the imaging session.
11. Vital signs monitoring during imaging was conducted using a SpO_2_ pulse oximeter and temperature rectal probe (Harvard Apparatus, USA).

### Intravital Imaging of Antigen-Specific T-Cells

Live imaging of OT-I^+^ CD8^+^ GFP^+^ T-cells within tumors was conducted on Day 21 on mice from all treatment cohorts (*n* = 6 mice per group from three independent experiments). Mice were immediately imaged after surgical preparation of skin-flap tumors on a Zeiss LSM 7 MP system equipped with W Plan-APOCHROMAT 20X/1.0 NA objective and Zeiss ZEN image acquisition software (Carl Zeiss Ltd, Germany). Fluorescence excitation and emission was achieved using a tunable titanium:sapphire two-photon source (Chameleon Ultra II, Coherent Laser Group, USA).

1. The appropriate laser wavelength (720–930 nm) and laser power (18%) were first set.
2. Epifluorescence illumination (pE-300^white^ LEF illumination unit, CoolLED Ltd, UK) was used to locate the tumor via the microscope eyepiece and a random tissue region where GFP^+^ T-cells and mCherry^+^ tumor cells were clearly visualized and selected for imaging.
3. The live imaging and second harmonic generation (SHG) modes were turned on and the focus was adjusted to locate collagen fibers in the tumor periphery. Adjustment to the upper and lower limits of the image stack were made: the upper limit or first imaging slice was set at a point where the SHG signal was just about to disappear, whilst the lower limit of the z-series was set by moving the objective to the maximum imaging depth (100–120 μm).
4. 3D time-lapsed images were acquired with an in-plane (*xy*) pixel resolution of 512 × 512 μm at increments of 5–6 μm in the *z* plane and a frame interval of 30 s. Recording time was ~30 min per scan.
5. The emitted light and SHG signals from the T-cells, tumor cells and collagen fibers were, respectively, collected with the bandpass filters for green (BP 520/60), red (BP 607/70) and second harmonic generation (SHG) (BP 447/60) and non-descanned PMT detectors.
6. Imaging was repeated at two other random regions of the tumor up to a total maximum period of 2 h in each mouse.
7. At the end of the intravital imaging experiment, the mouse was euthanized in a CO_2_ chamber. Once the breathing ceased, cervical dislocation was performed to ensure complete euthanasia.

### Flow Cytometry

Whole tumors with intact stroma were harvested from the mice after intravital imaging for subsequent functional analysis on flow cytometry. Single-cell suspension were prepared from tumors of no more than 1 g using gentleMACS Tumor Dissociation Kit (Miltenyi Biotec, Germany) and 40 μm cell strainers (Falcon^TM^, Thermo Fisher Scientific, USA). 5 × 10^6^ cells were incubated with Fc-block (eBioscience, USA) and stained in 2% FACS buffer at 4°C for 30 min with fluorochrome-conjugated antibodies (Biolegend, USA) against immune markers including CD45-BUV395 (clone 30-F11), CD8a-APC (clone 53-6.7), CD44-PerCPCy5.5 (clone IM7) and PD-L1-PE (clone 10F.9G2), as well as fixable viability dye eFluor 780 (Thermo Fisher Scientific, USA) for dead cells discrimination. Flow cytometry was conducted on BD LSRFortessa^TM^ analyzer (BD Biosciences, USA), with appropriate lasers and filters, unstained and compensation controls. Data were analyzed using FlowJo^TM^ (Tree Star Inc., BD Biosciences, USA).

### Immunofluorescence

Tumors from 18 mice per treatment cohort were fixed in 4% paraformaldehdye, embedded in low-melting agarose (Sigma Aldrich, USA) and sectioned into 300 μm thick slices on a VT1000S vibratome (Leica Microsystems, Germany). Immunofluorescence staining of the tumor stroma was performed at 4°C overnight in 0.1% sodium azide and 5% bovine serum albumin in PBS using rabbit anti-mouse Fibronectin (clone ab2413, Abcam, UK) and goat anti-rabbit Alexa Fluor 405 secondary antibodies (Molecular Probes, Life Technologies, USA); whilst tumor blood and lymphatic vessels were stained using rabbit anti-CD31 conjugated to Alexa Fluor 647 (clone MEC13.3, Biolegend). Stained sections were washed three times, mounted on custom-made deep well-microscope slides, and imaged in 10 random field-of-views on Zeiss 780 confocal microscope (Carl Zeiss Ltd, Germany).

### Image Processing and Analysis

Images were processed and analyzed using Icy, an open-source platform for bioimage analysis (32) and integrated with ImageJ version 1.52e (National Institute of Health, USA). All images were maximum intensity projected and attenuation corrected using the Debleach plugin, followed by Gaussian denoising and intensity thresholding for the detection of individual GFP^+^ T-cells.

#### Analysis of Immunofluorescence Images

The Edge Detection and Spot Detector plugins from the Icy software package were used for the semi-automated detection of the number of T-cells on immunofluorescent images. Histogram analysis was performed on the binary mask of the grayscale images to evaluate the spatial distribution of T-cells within the tumor microenvironment based on the individual T-cell GFP signal location in the *xy* coordinates horizontally from the tumor periphery toward the tumor core using Matlab (Mathworks, USA). First order statistical measure of image heterogeneity was computed based on the histogram kurtosis. Kurtosis is a measure of the peakedness in the distribution of gray values around the mean value (heavy-tailed or light-tailed relative to a normal distribution) i.e., heterogeneity of T-cell GFP signal distribution from tumor periphery to tumor core. Higher (positive) kurtosis reflects T-cells that are distributed more toward the tumor core in the immunofluorescent images.

#### Analysis of Time-Lapsed Videos

Time-lapsed videos from intravital imaging were corrected for motion artifacts or drifts using the Rigid registration plugin of the Icy software package. Tracking of individual T-cells was performed semi-automatically using the Spot Detector plugin for T-cell centroid identification and Manual Tracking plugin to determine the mean velocity, displacement and meandering index. T-cells that could not be tracked for more than 5 timepoints were excluded from the analysis. T-cell velocity was calculated from the straight-line distance between the point of origin and the point of termination of a cell track, divided by the duration of the cell track in minutes. The meandering index defines track straightness and is the ratio between the T-cell displacement from its origin and the total path length traveled, where 1 represents a completely linear cell track or directional movement (33). Track plots were calculated to demonstrate the actual migration of T-cells relative to their point of origin. The Edge Detection and Active Contour Icy software package plugins were used for semi-automated region of interest (ROI) segmentation of cell boundary to evaluate the dynamic changes in T-cell morphology over the imaging timepoints based on the measurement of cell elongation (34).

### Statistical Analysis

Statistical analysis was performed with Prism 5.01 (GraphPad Software Inc., USA). Data are presented as mean ± standard error of the mean (SEM) unless otherwise stated. Normality was assessed using the D'Agostino-Pearson normality test. Differences between two independent groups were evaluated using unpaired *t*-test for measurements that are normally distributed, Mann–Whitney test for data that are non-Gaussian. For comparison of three or more independent groups, the Kruskal–Wallis test followed by Dunn's multiple comparison *post hoc* test was used. A *p* < 0.05 was considered statistically significant.

## Results

### Development of a Syngeneic Mouse Melanoma Model for Immunotherapy and T-Cell Intravital Imaging

**Figure 1:**
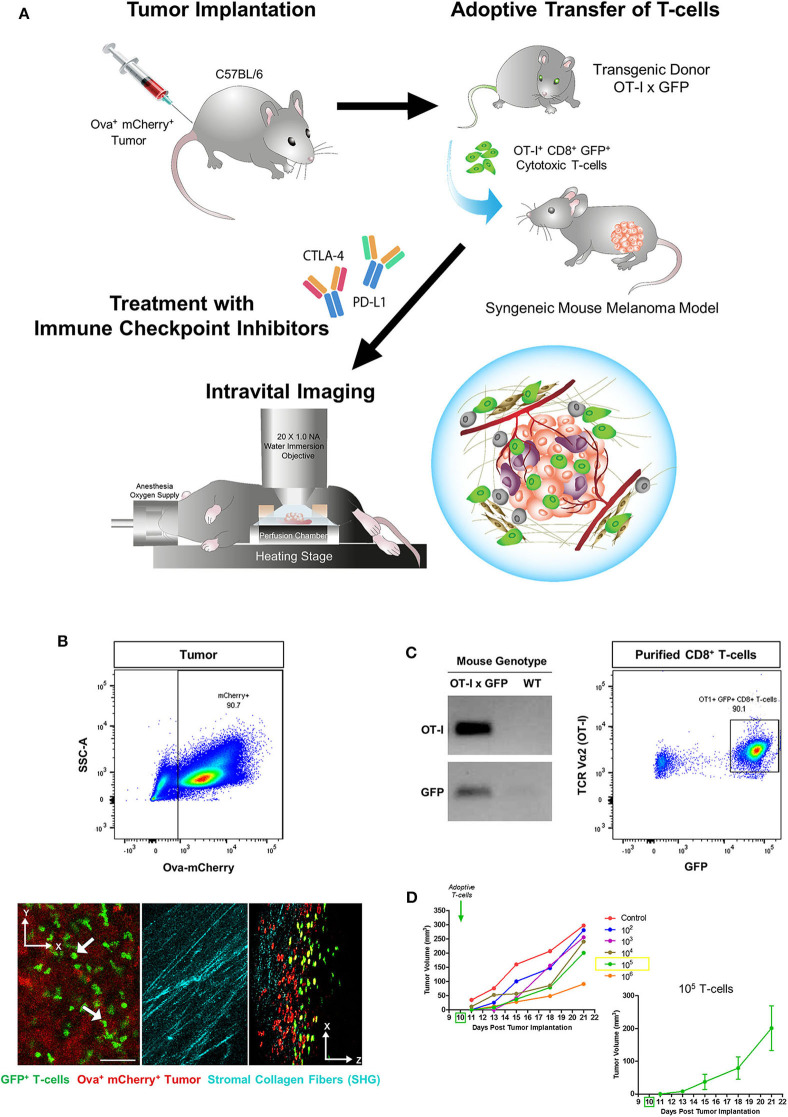
Generation of a syngeneic mouse melanoma for immunotherapy and T-cell intravital imaging. **(A)** OT-I^+^ CD8^+^ GFP^+^ T-cells were isolated from the lymph nodes of OT-I x GFP mice and adoptively transferred into C57BL/6 immunocompetent mice 10 days after subcutaneous implantation of B78ChOva-mCherry mouse melanoma tumors. Mice were treated with immune checkpoint inhibitors or vehicle control over 1 week and intravital imaging was conducted on Day 21. **(B)** The implanted tumors were amelanotic and expressed ovalbumin and mCherry for OT-I T-cell targeting and localization of the tumor-stroma interface by second harmonic generation imaging of collagen fibers. **(C)** Purified CD8^+^ T-cells for the adoptive T-cell transfer experiments were positive for both OT-I and GFP based on PCR genotyping and flow cytometry. **(D)** Mean tumor growth rates over 21 days of mice infused with titrated doses of OT-I^+^ CD8^+^ GFP^+^ T-cells on Day 10 (*n* = 6 mice for each titration group). Tumor volume changes at each timepoint following administration of 10^5^ T-cells are shown as mean ± SEM. The scale bar in **(B)** represents 50 μm. Graphics in **(A)** were original art illustrated by the author (DL).

A schematic diagram for the generation of a syngeneic mouse melanoma model used in these T-cell intravital imaging experiments is shown in Figure 1A. B78ChOva-mCherry is an amelanotic clone derived from the immunologically cold mouse B16 melanoma cell line. It is an ideal syngeneic melanoma model for intravital imaging and immunotherapy for the following reasons: the presence of the BRAFV600E mutation in B78 murine melanoma is reflective of ~50% of human malignant melanomas which have activating BRAF mutations; up to 20% of human melanoma are amelanotic or partially pigmented, and have poorer prognosis than pigmented tumors (35). The lack of melanin production in B78 due to a mutation in the tyrosinase-related protein 1 gene, minimizes autofluorescence in the green and red channel during optical imaging and enables better visualization of the dynamics of live GFP^+^ T-cell movement at the tumor site. Some studies have suggested that B78 is a weakly immunogenic tumor similar to its melanotic counterpart B16 melanoma, and immunotherapy such as vaccination strategies have been shown to enhance immune response in B78 (36, 37). B78ChOva-mCherry expresses the foreign antigen chicken ovalbumin that enabled T-cell targeting of the tumor and an mCherry reporter for visualization of the tumors. SHG imaging of fibrillar collagen and myofibers allowed location of the tumor periphery during imaging (Figure 1B). Cross-breeding of OT-I mice and GFP mice generates CD8^+^ T-cells with cell membrane expression of the T-cell receptor comprising the alpha (Vα2) and beta (Vβ5) chains of OT-I that can specifically recognize ovalbumin (30, 38). Strong ubiquitous expression of the GFP reporter in the cytoplasm enabled measurements of T-cell motility, dynamic changes in cell morphology and distribution within the solid tumor microenvironment (31), without the need for additional antibody labeling during live imaging.

T-cells purified from the lymph nodes of OT-I x GFP mice were typically more than 90% positive for both OT-I and GFP (Figure 1C). As the infusion dose of antigen-specific T-cells can have an impact on the magnitude of tumor regression, as well as the number of viable tumor cells and T-cells that could still be detected on Day 21 during intravital imaging, initial experiments were performed to adoptively transfer titrated doses of OT-I^+^ CD8^+^ GFP^+^ T-cells into mice bearing established B78ChOva-mCherry tumors on Day 10. Infusion with increasing numbers of T-cells caused a greater delay in tumor growth compared to lower numbers of injected cells (Figure 1D). Infusion with 10^5^ T-cells was selected for subsequent drug treatment and imaging experiments as tumor growth was slower than at lower T-cell doses but not as severely delayed at Day 21 compared to infusion with 10^6^ T-cells.

### Immune Checkpoint Inhibition Enhanced T-Cell Recruitment and Activation

**Figure 2:**
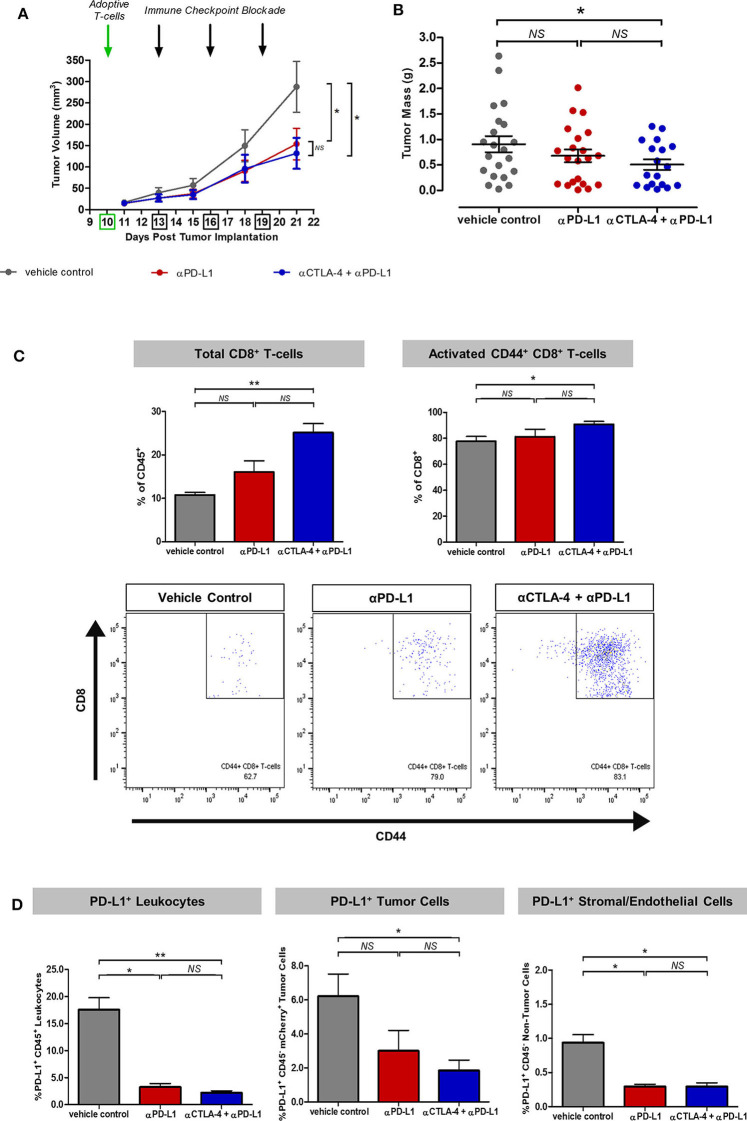
Synergistic effects of immune checkpoint inhibitors on T-cell recruitment and activation in the experimental model for intravital imaging. **(A)** Tumor growth rates of mice treated with adoptive T-cell transfer alone (vehicle control) or in combination with monoclonal antibodies targeting PD-L1 or CTLA-4. **(B)** Tumor mass harvested at the end of the intravital imaging experiment on Day 21. **(C)** Flow cytometric analysis of harvested tumors showed enhanced recruitment and activation of adoptive and endogenous T-cells based on the expression of the CD8 marker for cytotoxic T-cells and the CD44 marker for T-cell migration, activation and effector/memory response. **(D)** Effects of immune checkpoint inhibitors on PD-L1^+^ immune and non-immune components of the tumors across treatment cohorts. Unpaired *t*-test was performed to test for differences in tumor volumes and tumor masses on Day 21 between ICI-treated cohorts and the vehicle control **(A,B)**. Kruskal–Wallis test with *post-hoc* Dunn's multiple comparison analysis was performed to test for differences between all three independent treatment cohorts **(C,D)**; *P < 0.05; **P < 0.01.

To demonstrate the effects of immune checkpoint inhibitors on adoptive T-cell response in this experimental model for intravital imaging, we first administered 10^5^ OT-I^+^ CD8^+^ GFP^+^ T-cells on Day 10 and allowed the transgenic T-cells to be reconstituted into the wild-type mouse immune system for at least 48 h before treatment from Day 13 with monoclonal antibodies targeting PD-L1 and/or CTLA-4 every 2–3 days over the course of 1 week. Rapid exponential tumor growth from Day 10 was observed in mice given adoptive T-cell transfer alone (mean tumor volume 288 mm^3^). By Day 21, combined therapy with immune checkpoint inhibitors (ICI) resulted in slower tumor growth rates. Combined adoptive T-cell transfer with anti-CTLA-4 and anti-PD-L1 therapy (132 mm^3^) was only marginally more effective than anti-PD-L1 monotherapy (144 mm^3^) (Figure 2A). The mass of tumors harvested at the end of the imaging experiment on Day 21 was more variable in all treatment cohorts, but also suggested combination therapy was more effective than monotherapy (Figure 2B).

Flow cytometric analysis of the harvested tumors on Day 21 after intravital imaging showed a greater percentage of endogenous and adoptive CD8^+^ T-cells infiltration within ICI-treated tumors (*p* < 0.01 for vehicle control vs. combined anti-CTLA-4 and anti-PD-L1). Expression of the T-cell migration, activation and effector/memory response marker CD44 was higher in tumors treated with combined anti-CTLA-4 and anti-PD-L1 therapy (*p* < 0.05), compared to control mice (Figure 2C). Further analysis revealed an overall reduction in PD-L1^+^ cell populations in ICI-treated tumors, which can be accounted for by immune-mediated cell death (*p* < 0.05 for vehicle control vs. anti-PD-L1 monotherapy; *p* < 0.01 for vehicle control vs. combined anti-CTLA-4 and anti-PD-L1; Figure 2D).

### Greater T-Cell Infiltration and Heterogenous Distribution in Experimental Model

**Figure 3:**
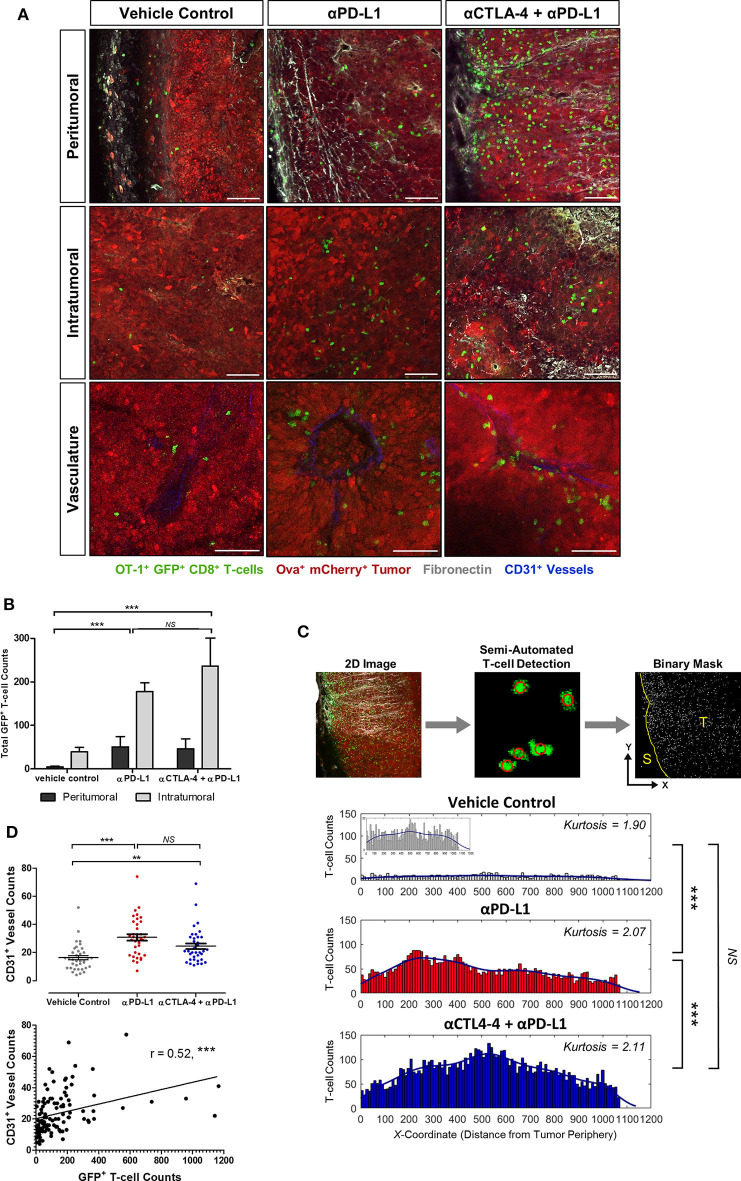
Demonstration of the enhanced infiltration and heterogeneous distribution of adoptive T-cells in tumors treated with immune checkpoint inhibitors. Immunofluorescence analysis was performed on harvested tumors obtained on Day 21 of the experiment and 11 days after adoptive T-cell transfer and 8 days after immune checkpoint inhibition was initiated (*n* = 18 mice per treatment cohort). **(A)** Spatial distribution of OT-I^+^ CD8^+^ GFP^+^ T-cells in tumors across treatment cohorts. **(B)** Image channel with GFP^+^ signal was extracted from maximum-intensity projected immunofluorescence images (300 μm slice thickness; 10 fields-of-view taken horizontally from stroma, invasive margin and toward tumor core). Edge Detection and Spot Detector plugins were used for automated detection of GFP^+^ T-cells. Binary masks of the T-cell *xy* coordinates were used to calculate total GFP^+^ T-cells counts in peritumoral and intratumoral regions of the tumors and **(C)** the histogram analysis on the spatial distribution of adoptive T-cell within the solid tumors. **(D)** CD31^+^ vessel counts (mean ± SEM) and Spearman's correlation analysis of CD31^+^ vessel counts and GFP^+^ T-cell counts. Mann–Whitney test was performed for testing differences in total GFP^+^ T-cell counts between the vehicle control and ICI-treated cohorts **(B)**. Kruskal–Wallis test with *post hoc* Dunn's multiple comparison analysis was performed to test for differences between three independent treatment cohorts **(C,D)**; **P < 0.01; ***P < 0.001. Scale bars in **(A)** represent 100 μm.

To evaluate how immune checkpoint inhibitors targeting CTLA-4 and PD-L1 improve adoptive T-cell migration into tumors in this experimental model, image-based analysis of the spatial distribution of the OT-I^+^ CD8^+^ GFP^+^ T-cells in the harvested tumors was performed (*n* = 18 mice from each treatment cohort). A greater number of GFP^+^ T-cells were detected in both peritumoral and intratumoral regions of tumors treated with ICI, compared to tumors treated with T-cell transfer alone (*p* < 0.001; Figures 3A,B). Histogram analysis showed a more heterogeneous spatial distribution of adoptive T-cells within ICI-treated tumors (kurtosis = 2.07 for PD-L1 monotherapy; 2.11 for combined anti-CTLA-4 and anti-PD-L1 therapy) compared to tumors treated with T-cell transfer alone (kurtosis = 1.90). More T-cells were detected at the invasive margins and toward the tumor core in tumors treated with combined anti-CTLA-4 and anti-PD-L1 therapy (Figure 3C). The pan-endothelial cell marker CD31 is expressed on blood and lymphatic vessels (39): higher CD31^+^ vessel counts were detected in ICI-treated tumors than in the control mice. Spearman's correlation analysis showed a positive relationship between GFP^+^ T-cell numbers and CD31^+^ vascular density (Figure 3D).

### Distinct Adoptive T-Cell Behavior and Cellular Morpho-Dynamic Changes

Intravital imaging using the methodology described in B78ChOva-mCherry was performed to study the *in vivo* behavior and cellular morpho-dynamics of adoptive T-cells. Immune checkpoint blockade was used as an example to demonstrate this approach. Individual GFP^+^ T-cells could be resolved and tracked up to the penetration depth limit of 100 μm beneath the tumor periphery of a skin-flap tumor model.

**Figure 4:**
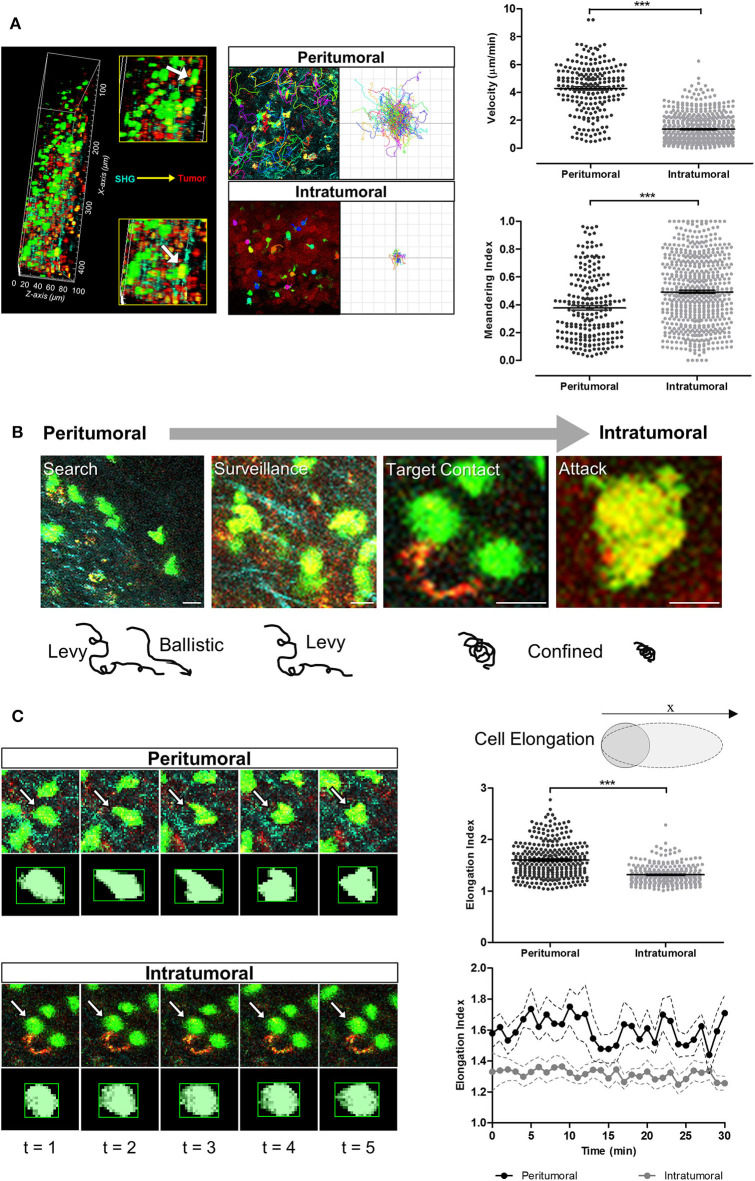
T-cell migration speeds, trajectories and cellular morpho-dynamic changes during tumor infiltration. **(A)** Intravital imaging was performed using multiphoton microscopy with second harmonic generation to detect the stroma-tumor interface and OT-I^+^ CD8^+^ GFP^+^ T-cells infiltrating tumors at a tissue penetration depth of 100 μm. Track plots, velocities, and meandering indices of T-cells were measured from T-cells located separately within the peritumoral and intratumoral regions. **(B)** Adoptive T-cells demonstrated different migration patterns from peritumoral to intratumoral regions of the tumors. **(C)** Analysis of T-cell morphology changes over the 30 min imaging time-course. Mann–Whitney test **(A,C)** for differences between the means of two independent groups; ***P < 0.001. Scale bars in **(B)** represent 10 μm.

The majority of T-cells at the tumor periphery were observed moving slowly along the collagen fibers with Lévy-like trajectories consisting of clusters of small steps interspersed with long walks (40). T-cells migrating in the collagen-dense areas of the tumor periphery demonstrated significantly faster movements (4.27 ± 0.11 μm/min) than T-cells within the tumor core (1.36 ± 0.04 μm/min; *p* < 0.001; Figure 4A). These peritumoral T-cells displayed more elongated lamellipodia at the leading edge and shortened uropods at the rear end with more rapidly changing morphology (elongation index of 1.60 ± 0.01) and less meandering or confined movements (meandering index of 0.37 ± 0.01) than T-cells in the tumor core (elongation index of 1.31 ± 0.01; meandering index of 0.49 ± 0.01; *p* < 0.001). In contrast, intratumoral T-cells exhibited a more compact morphology with dynamic formation and contraction of filopodia as they navigated the tumor core. Some T-cells in close contact with the tumor cells were stationary, rounded and appeared to undertake probing behavior in the local environment, consistent with cell-cell interactions (Figures 4B,C).

**Figure 5:**
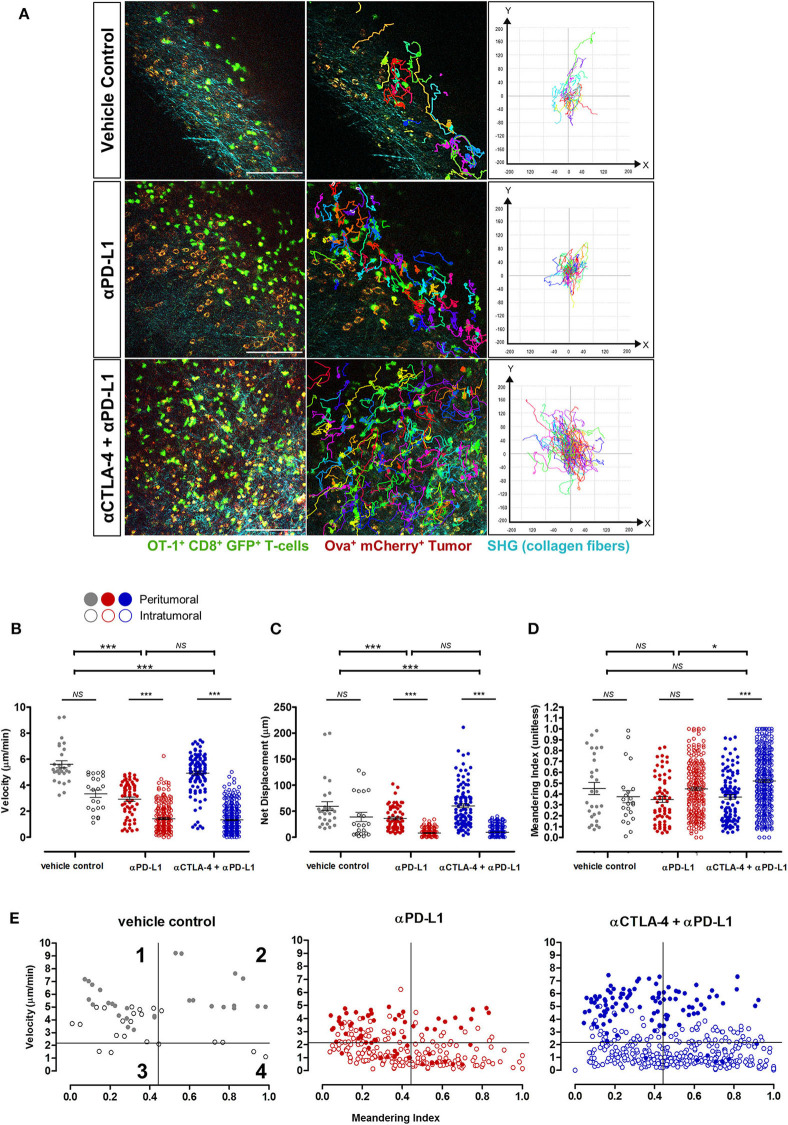
*In vivo* behavior of adoptive T-cells in response to immune checkpoint blockade. **(A)** Representative images from Supplementary Videos 1–3 and track plots of OT-I^+^ CD8^+^ GFP^+^ T-cells from both peritumoral and intratumoral regions of tumors across treatment cohorts. **(B)** Velocity, **(C)** displacement, and **(D)** meandering index of all T-cells from the different treatment cohorts (*n* = 6 mice per cohort). **(E)** Individual T-cell tracks were plotted according to their meandering indices and mean velocities for qualitative analysis of the different migration behavior patterns of T-cell populations in tumors of mice given different treatments, and quadrants on the plots depicts four populations of T-cells (42–44): (1) actively migrating with return to their origins; (2) directional and sustained movements; (3) low motility; and (4) T-cells with non-sustained motility (see Results for further explanation of these terms). The Kruskal–Wallis test with *post hoc* Dunn's multiple comparison analysis **(B–D)** for differences between three independent treatment cohorts was used for statistical analysis; *P < 0.05; ***P < 0.001. Scale bars in **(A)** represent 100 μm.

Adoptive T-cells exhibited diverse migration patterns in the tumors of mice across all treatment cohorts. More T-cells were detected in the tumor periphery and tumor core of mice treated with ICIs, and were observed traveling at significantly lower mean velocities (1.75 ± 0.07 μm/min for anti-PD-L1 monotherapy; 2.15 ± 0.08 μm/min for combined anti-CTLA-4 and anti-PD-L1 therapy) than T-cells from the vehicle control cohort (4.60 ± 0.25 μm/min; *p* < 0.001; Figures 5A–D). T-cells in ICI-treated tumors exhibited a much shorter displacement from their original position (14.38 ± 0.99 μm for anti-PD-L1 monotherapy; 21.32 ± 1.38 μm for combined anti-CTLA-4 and anti-PD-L1 therapy; *p* < 0.001) and more confined movements (meandering index of 0.42 ± 0.01 for anti-PD-L1 monotherapy and 0.47 ± 0.01 for combined anti-CTLA-4 and anti-PD-L1 therapy; *p* < 0.05) than T-cells within tumors of mice from the vehicle control group (mean displacement of 50.78 ± 6.35 μm; meandering index of 0.41 ± 0.03; Figure 5D; Supplementary Videos 1–3). T-cell velocities were higher in the peritumoral region compared to intratumoral region in mice from all treatment cohorts.

Qualitative analysis of the migration behavior patterns of T-cell populations in the tumors of mice given different treatments were performed by plotting the mean velocities vs. the meandering index of individual T-cell migration tracks (Figure 5E) (41–43). For further comparison on the differences in T-cell behavior across treatment cohorts, each migration plot was subdivided into quadrants based on the mean velocity (2.16 μm/min) and meandering index (0.45) of all adoptive T-cells measured in tumors of mice from all treatment cohorts. Each numbered quadrant on the migration plots represents different T-cell migration patterns within the solid tumor microenvironment: (1) actively migrating T-cells returning to their origin i.e., high mean velocities and low meandering indices; (2) T-cells with directional and sustained movements i.e., fast movements with minimal stop periods as reflected by the high mean velocities and high meandering indices; (3) T-cells with low motility i.e., low mean velocities and low meandering indices; and (4) T-cells with non-sustained motility i.e., low mean velocities and but high meandering indices which implied that these T-cells have more prolonged periods of immobility and only actively migrate during certain periods of the observation. Adoptive T-cells in ICI-treated mice exhibited variable migration patterns, with cells distributed in all four quadrants of the T-cell behavior distribution. An enriched number of intratumoral T-cells were observed in quadrants 3 and 4, implying the presence of more T-cells with non-sustained and low mobility with short periods of active movements, consistent with the behavior of activated T-cells engaging tumor targets. A higher number of fast-moving T-cells (both with directional/sustained migration and with a low meandering index i.e., quadrants 1 and 2 above) were observed in tumors of mice treated with combined anti-CTLA-4 and anti-PD-L1 therapy compared to anti-PD-L1 monotherapy. The majority of these fast-moving T-cells in ICI-treated mice were in the peritumoral region.

## Discussion

Efficient T-cell infiltration, homing, and activation within solid tumors is crucial for the success of cancer immunotherapy. Increasing evidence suggests that T-cell migration into peripheral tissues is not random, but governed by a combination of cell-intrinsic locomotion events (driven by actin polymerization and myosin-induced contractions), physical guidance from the microenvironment including the stroma, chemical cues (such as cytokines, chemokine gradients), the density of cognate antigens and antigen-presenting cells (APCs), as well as the presence of co-stimulatory molecules and immune checkpoints (44, 45). In solid cancers, T-cells are frequently found at greatest density in peritumoral regions and poorly penetrate the dense tumor (12, 45). The high density of regulatory T-cells (Tregs) and myeloid-derived suppressor cells (MDSCs) that are recruited during tumorigenesis, as well as the presence of intrinsic cancer-associated fibroblasts, have been shown to reduce the T-cell response through the expression of PD-L1 and other immunosuppressive molecules (6, 8). Live imaging of T-cell behavior in the context of tumor immunosuppression and following pharmacological intervention with immuno-modulatory drugs could have important implications for understanding the interaction of T-cells with the tumor and its environment, as well as studying the functional effect of novel therapies such as ATC in conjunction with immuno-modulatory drugs.

Intravital imaging has proven to be an important tool for understanding the effects of cancer immunotherapy on tumor eradication (24). The *in vivo* dynamics of single T-cell behavior, function, and its complex interactions with the solid tumor microenvironment can be studied at high spatial resolution that is not easily achievable by *in vitro* assays and other macroscopic imaging techniques such as MRI and PET (26, 27). Several experimental approaches to intravital imaging can be utilized for studying T-cell behavior following immunotherapy in living animals (24, 25): abdominal and dorsal skin-fold window chambers are available for longitudinal tracking of T-cell dynamics (46, 47); small tumors can be implanted into the ear pinnae or footpad for non-invasive imaging (48); skin-flap tumors can be surgically exposed to gain immediate insights on T-cell dynamics following treatment. Each of these methods has advantages and limitations and the choice of method depends not only on the biological question being addressed, but also on the extent of technical expertise and equipment required to carried out the procedure. The use of a skin-flap tumor model for intravital imaging is limited by the need for surgical exposure of the tumors for fluorescent imaging. However, unlike the dorsal skin-fold window chamber or ear tumor models, skin-flap tumors are essentially subcutaneous implanted tumors allowed to grow in all dimensions in a similar manner to the tumor models used in most immunological studies. As tumors have limited spaces for growth within window chambers or between the ear dermis, it may be difficult to control the size of tumor growth and develop a larger established tumor for assessing immunotherapy. Clamping of the tissues in a dorsal skin-fold chamber and growing the tumor within the confined space of the ear dermis can disrupt the normal surrounding tissues and limit blood perfusion (49). The window chamber and ear tumor models are also technically challenging for most researchers, as complex surgery is required to remove normal tissues to implant the artificial window and tumor spheroids. This poses a high risk for tissue inflammation and animal loss, hence careful monitoring during the post-surgical recovery phase would be required even before the start of any treatment. Nevertheless, the main advantage of window chamber and ear tumor models is the opportunity to undertake longitudinal imaging.

In this study, a combination of intravital imaging followed by detailed image and functional analysis was performed *in situ*, to reveal insights on the function and behavior of adoptive T-cells in skin-flap tumors. Treatment with CTLA-4 and PD-L1 monoclonal antibodies was used as an exemplar to demonstrate this experimental approach. In comparison to the untreated control group, tumors treated with checkpoint inhibitors demonstrated more adoptive T-cell infiltration at the invasive margins at the junction between the collagen fibers detected with second harmonic generation, and the mCherry-labeled tumors, as well as the upregulation of CD44, an adhesion marker which mediates T-cell migration into the tumor interstitium (41). Treatment with anti-PD-L1 monotherapy resulted in a substantially lower tumor mass compared to tumors treated with T-cell transfer alone, as well as increased depletion of PD-L1^+^ tumor cells, leukocytes, stromal and endothelial subpopulations within the tumor microenvironment at Day 21.

We had used this experimental setup to show that adoptively transferred T-cells are spatially heterogeneous within tumors. T-cells were recruited mainly to the stromal regions within tumors, with some zones completely devoid of T-cell infiltration. A similar spatial heterogeneity has been previously observed at a macroscopic level using MRI cell tracking methods, in which significant intratumoral heterogeneity in T2 signal has been reported in a B16-OVA melanoma model adoptively transferred with OVA-specific CD8^+^ T-cells labeled with iron oxide nanoparticles (50). In our study, adoptive T-cells were recruited in greater numbers toward the tumor core following immune checkpoint inhibition. This observation at the microscopic level is in concordance with previous PET studies using anti-CD8 radiolabeled antibodies in which more intratumoral tracer uptake was observed in B16.F10 melanoma tumors that responded to CTLA-4 therapy, whilst the non-responders displayed a more peripheral distribution in the detected signal (51). In a separate study, higher intratumoral tracer was observed in BALB/c mice bearing CT26 colorectal tumors responding to PD-L1 therapy, whilst the non-responders displayed a peripheral rim of activity (52). The proximity of CD8^+^ T-cells to the tumor cells and the greater distribution toward the tumor core seems to be an indicator of tumor control and treatment response. These findings at both microscopic and macroscopic scales resemble human solid cancers, in which CD8^+^ cell infiltration at the invasive margin has been observed in melanoma patients responding to anti-PD-1 therapy (45).

Both CTLA-4 and PD-1/PD-L1 signaling pathways have been implicated in many aspects of T-cell function, motility and migration into peripheral tissues. CTLA-4 is a negative regulator of T-cell mediated immune response and acts predominantly during the priming phase of early T-cell activation following antigen encounter in secondary lymphoid organs (12). Expression of CTLA-4 on T-cells upon TCR activation can compete with the co-stimulatory receptor CD28 for binding to B7 ligands and dampens CD28 signaling which is usually required for activating downstream processes involved in the regulation of cytoskeletal reorganization and integrin-mediated T-cell adhesion during tissue infiltration, such as phosphoinositide-3 kinase (PI3K) and small Rho GTPase Cdc42 signaling (53, 54). CTLA-4 has also been shown to interfere with T-cell activation by disrupting the process of antigen-presentation by APCs in the lymph nodes and the formation of an immunological synapse through increased T-cell motility and limited dwell times around dendritic cells, as proposed by the “reverse-stop signal” mechanistic model of CTLA-4 inhibitory function (44, 55). This was further evidenced by reported cases of systemic lymphoproliferation and enhanced T-cell infiltration into tumors and other non-lymphoid organs in CTLA-4 knockout mice and both human and mice treated with anti-CTLA-4 (12, 44). Similarly, PD-1/PD-L1 signaling has been known to inhibit T-cell activation and acts predominantly on effector CD8^+^ T-cell function. CTLA-4 and/or PD-L1 blockade in tumors has been demonstrated to act via the depletion of regulatory and immunosuppressive cell types such as Tregs and MDSCs and reinvigorating exhausted T-cells to facilitate tumor entry for more effective tumor eradication (6, 17). The exact mechanism by which PD-1/PD-L1 signaling inhibits T-cell entry into tumors has yet to be elucidated and may be tissue-dependent. PD-1/PD-L1 signaling has been known to suppress PI3K activities downstream of CXCR5 signaling in activated CD4^+^ T-cells and inhibit their recruitment into the splenic lymphoid follicles for follicular T helper cell development during T-cell dependent B-cell response (56). In contrast, therapeutic blockade of PD-1 and PD-L1 has been shown to restore T-cell motility in the presence of high viral loads during the early stages of chronic lymphocytic choriomeningitis viral infections in mice (57). As CTLA-4 and PD-L1 are implicated in many aspects of T-cell biology, and monoclonal antibodies targeting these two immune checkpoint proteins are clinically available (12, 17), these pathways could be potentially exploited to improve the efficacy of adoptive T-cell therapy in solid tumors.

Live imaging of adoptive T-cells performed here has revealed insights on the morpho-dynamics of T-cell within the solid tumor microenvironment with and without ICI treatment. T-cells at the tumor periphery traveled at higher velocities in a mixed Lévy and ballistic-like random search mode along the collagen fibers, with straighter trajectories and rapidly changing cell shape and elongation in the direction of travel, compared to T-cells at the tumor core. This pattern of movement is consistent with a tumor seeking trajectory: Lévy-like movements maximize the chances of T-cells encountering their targets and have been observed in a number of biological scenarios, ranging from the ecological behaviors of animals foraging for food to metastatic cancer cells migration at the microscopic level (40, 58, 59). T-cells located near the invasive margin and toward the tumor core also exhibited some Lévy-like locomotion but demonstrated more confined trajectories; these cells were more compact in shape compared to those in the periphery, and this morphology and pattern of movement may reflect cells that have already engaged their targets. Following treatment with immune checkpoint inhibitors, more T-cells were detected traveling at lower mean velocities and with shorter displacement and more meandering tracks compared to control mice which may also reflect established cell-cell interactions. T-cells exhibited a range of migration patterns within the solid tumor microenvironment and mostly displayed low motility and confined movements within the ICI-treated tumors at Day 21 (~11 days after adoptive T-cell transfer and 8 days after the start of ICI treatment). This low motility and confined movement suggested that the T-cells may have identified their antigen target and that cytotoxic killing may be in progress. Adoptive T-cells were observed moving more rapidly in the tumors of mice treated with combined anti-CTLA-4 and anti-PD-L1 therapy, compared to anti-PD-L1 monotherapy. Here we have identified four distinct subpopulations of T-cells based on their migration behaviors with combinations of high vs. low motility, and high vs. low meandering index. After CTLA-4 and PD-L1 blockade, there was an enrichment of T-cells with low motility and high meandering, implying that these T-cells were rapidly infiltrating into the solid tumor microenvironment to search for antigen targets. In comparison to PD-L1 monotherapy, combined CTLA-4 and PD-L1 blockade resulted in only moderate treatment effects (though not statistically significant) on tumor shrinkage and T-cell activation and migration i.e., a greater number of GFP^+^ T-cells and populations of cells with higher velocities were measured in tumors given both antibodies. CTLA-4 and PD-L1 are known to be expressed on Tregs and melanoma cells. Treatment with blocking antibodies can lead to depletion of these two immunosuppressive cell types within the tumor microenvironment, in addition to the re-activation of exhausted cytotoxic T-cells (60, 61). The results from our preliminary analysis in this methods paper suggest that although the combined effect of CTLA-4 and PD-L1 monoclonal antibodies is synergistic, targeting PD-L1 alone may be sufficient. Although changes in T-cell proliferation and survival in the timepoint analyzed may have contributed to the increase in T-cell infiltration, the effects of CTLA-4 and PD-L1 antibodies treatment on tumor growth and T-cell function was still evident i.e., tumors undergoing both monotherapy and combined therapy were approximately half the volume of the controls and the number of GFP^+^ T-cells was 3–5-fold higher in both the peritumoral and intratumoral regions of the ICI-treated mice compared to the controls. Further mechanistic studies using this experimental model will be useful for understanding the effect of immune checkpoint inhibitors on T-cell behavior in solid tumors.

In summary, intravital imaging using a skin-flap tumor model and adoptive transfer of green fluorescent T-cell has demonstrated the feasibility of this experimental approach for direct visualization of antigen-specific T-cell function and behavior within the solid tumor microenvironment, as well as changes in response to immuno-modulatory drugs. Evaluation of T-cell motility in this experimental model following treatment with CTLA-4 and PD-L1 targeted immune checkpoint inhibitors demonstrated diverse patterns of T-cell behaviors including change in shape, mean velocities, and trajectories.

## Conclusion

In this study, we have demonstrated the feasibility of using intravital imaging and a transgenic ovalbumin-expressing mouse melanoma with adoptively transferred green-fluorescent OT-1^+^ cytotoxic T-cells to study antigen-specific T-cell function and behavior. This experimental approach provides immediate insights on changes in T-cell migration patterns during tumor infiltration and is a useful tool for understanding the effects of immuno-modulatory drugs such as immune checkpoint inhibitors on T-cell motility.

## Data Availability Statement

All datasets generated for this study are included in the article/Supplementary Material.

## Ethics Statement

The animal study was reviewed and approved by Animal Welfare and Ethics Review Committees of the University of Cambridge and The Babraham Institute in accordance with the United Kingdom Home Office's Guidance on the Operation of Animals (Scientific Procedures) Act 1986.

## Author Contributions

DL conducted all the experiments. FG and AC assisted DL with setup of experiments. DL, FAG, and KO designed the study, analyzed the data, and interpreted the results. DL, FAG, and KO wrote the manuscript. All authors contributed to the discussion, manuscript editing, read, and approved the final version before submission.

## Conflict of Interest

The authors declare that the research was conducted in the absence of any commercial or financial relationships that could be construed as a potential conflict of interest.

## Supplementary Material

Supplementary Video 1 Intravital imaging of T-cell behavior on Day 21 in a B78ChOva-mCherry tumor treated with adoptive T-cell transfer alone (vehicle control). Fast-moving T-cells were detected in the collagen-dense areas of the tumor periphery, with rapidly elongating cell shape during the time-course of imaging. T-cells are GFP^+^ (green), tumor cells are mCherry^+^ (red), and collagen fibers were seen as second harmonic signals (cyan).

Supplementary Video 2 Intravital imaging of T-cell behavior on Day 21 in a B78ChOva-mCherry tumor treated with PD-L1 monotherapy. An enriched number of GFP^+^ T-cells in the tumor periphery and tumor core were identified traveling at lower mean velocities, shorter displacement and with more confined trajectories in anti-PD-L1 treated tumors as compared to the controls. Some T-cells in close contact to the tumor cells were with rounded, compact cell shape and probing behaviors. T-cells are GFP^+^ (green), tumor cells are mCherry^+^ (red) and collagen fibers were seen as second harmonic signals (cyan).

Supplementary Video 3 Intravital imaging of T-cell behavior on Day 21 in a B78ChOva-mCherry tumor treated with combined anti-CTLA-4 and anti-PD-L1 therapy. An expansion in the numbers of GFP^+^ T-cells were detected in tumors of mice treated with combined immune checkpoint inhibitors therapy. High number of fast-moving T-cells with directional and sustained movement in the tumor periphery were detected, and numerous T-cells were observed with low motility and confined movements near tumor cells. T-cells are GFP^+^ (green), tumor cells are mCherry^+^ (red), and collagen fibers were seen as second harmonic signals (cyan).
